# Facile Liquid-Exfoliation Process of Boron Nitride Nanosheets for Thermal Conductive Polyphthalamide Composite

**DOI:** 10.3390/polym11101628

**Published:** 2019-10-09

**Authors:** Seokgyu Ryu, Hyunwoo Oh, Jooheon Kim

**Affiliations:** School of Chemical Engineering & Materials Science, Chung-Ang University, Seoul 06974, Korea; waw1991@naver.com (S.R.); ohwoo7@cau.ac.kr (H.O.)

**Keywords:** thermal conductivity, polymer composite, exfoliation

## Abstract

In this study, we describe the fabrication of thermally conductive composites based on a polyphthalamide (PPA) matrix by the exfoliation of hexagonal BN nanosheets (BNNs) via the melt-mixing method. Boron nitride (BN) particles were hydroxyl groups surface-treated with sodium hydroxide (NaOH). Compared with existing BN peeling experiments, we successfully produced BNNs that are simpler, more economical, and have an excellent aspect ratio. For the same weight content of BN and BNNs, PPA/BN composites surface-treated with high aspect ratio BNNs have a high in-plane and through-plane thermal conductivity because of the intercalation of the hydroxyl group surface treatments between BN and PPA, which not only increases the wettability but also provides a good heat transfer path. Moreover, wide and thin BNNs are evenly dispersed inside the PPA/BN composite to provide excellent heat transfer paths in both in-plane and through-plane directions.

## 1. Introduction

One recent advancement indicating the tremendous progress in the field of electronics is the miniaturization of transistors, which allows for the integration of a greater number of transistors into a single device and improves device performance. However, the rise in temperatures accompanied by this miniaturization is one engineering concern. The overheating of electronic devices can reduce the life of a device by more than half [1,2,3,4]. Therefore, in order to ensure durability and stability, it is essential to heat dissipation that it be integrated for the operation of next-generation electronic devices. The reason why the heat conductivity between the heat sink and the electronic device is low, is due to the interfacial characteristics of these two surfaces (i.e., interstitial gaps). Here, we show that by using a thermally conductive boundary material (TIM) to adhere to the rough interface structure, we can eliminate this crevice gap at the interface. Typically, TIM is used to induce excellent heat transfer by minimizing the pores between the two interfaces [5,6,7].

Among of super engineering plastics, polyphthalamide (PPA) has excellent heat resistance, melt stability, and mechanical properties (flexural modulus and flexural fatigue characteristics), and it is widely used as an external material for electronic devices.PPA has excellent heat resistance, melt stability, and mechanical properties (flexural modulus and flexural fatigue characteristics), and it is widely used as an external material for electronic devices. Additionally, PPA has excellent insulation properties, excellent molding processability, and also has excellent chemical properties. For these reasons, PPA serves as an excellent matrix for polymeric materials. Unfortunately, most PPAs tend to exhibit poor thermal conductivity (≈0.18 W m^−1^K^−1^). Several studies have focused on improving the thermal conductivity of PPA by filling cost-effective thermal fillers [8,9].

Thermally conductive fillers are primarily inorganic ceramics, such as alumina (Al_2_O_3_), magnesium oxide (MgO), silicon carbide (SiC), aluminum nitride (AlN), and BN. In addition, these also include carbonaceous materials, such as multiwalled carbon nanotubes (MWCNT), carbon fiber, graphene, and metallic materials such as copper (Cu) and aluminum (Al). Among these, the carbon-based materials exhibit the highest thermal conductivity. The theoretical thermal conductivity of MWCNTs is greater than 3000 W/(m·K), whereas that of graphene is greater than 2000 W/(m·K) [10]. These figures are far higher than those of inorganic ceramic fillers.

Unfortunately, these figures are only theoretical as there are limitations. When these materials are used as fillers in TIM, the thermal conductivity, as well as the electrical conductivity rises, which causes device malfunction in miniaturized devices. The rise in conductivity occurs equally with the use of a metal-based filler. Therefore, it is good to use ceramic fillers that have good electrical insulation and improve the thermal conductivity of the composite. Ceramic powders, such as aluminum nitride, BN, and silicon carbide, have excellent insulation, as well as high thermal conductivity and are suitable for use as a composite material. In this study, we focused on hexagonal BN as a filler because it has a price advantage and a high thermal conductivity. However, the surface of BN particles has strong interaction and cohesive properties. Moreover, the composite is not friendly to the surface of the polymer, and therefore, does not have an excellent effect when the composite is manufactured [11,12].

Therefore, many studies have been working on improving the interfacial affinity between BN particles and polymers. Moreover, the surface treatment of BN is not economical due to its weak result of polymer composites thermal conductivity and the time and high cost of surface treatment. Taking other studies as an example, the surface treatment of cured silane on BN resulted in attachment to only a single edge of h–BN and a very slight increase in thermal conductivity. However, polymer composites using BN nanosheets (BNNs) prepared by the exfoliation of BN in the form of a thin nanosheet, showed impressive increases in mechanical strength and thermal conductivity when their thickness and plate surfaces were controlled. The most widely used method for the exfoliation of BN is peeling using ultrasonication. Although these methods are simple due to the power of ultrasonication, the BN is peeled off and the plate is broken as well, which creates disadvantages in making BNNs with a wide plate shape. When combined with polymers, BNNs have a thinner and higher surface area with higher aspect ratios, and even higher thermal conductivity when combined with polymers. Therefore, the exfoliation of BN by ultrasonication, limits the production of excellent BNNs. For this reason, studies are underway to exfoliate thin nanosheets while minimizing the damage of BN flakes. In this study, a surface treatment of the hydroxyl group on the BN surface and a simple method of exfoliation without destroying particles of BN flakes were studied [13,14,15].

The PPA composites were fabricated using a twin extruder via a melt-mixing method. Structural differences were studied using Fourier transform infrared spectroscopy (FT-IR) and X-ray photoelectron spectroscopy (XPS) after NaOH-treated surface modification. We used atomic forces microscopy (AFM), transmission electron microscopy (TEM), and field emission scanning electron microscopy (FE-SEM) to identify the BNBN exfoliation. To investigate the effect of the improvement of the surface modification of PPA and BN on the thermal conductivity, a thermal conductivity analysis was performed using laser flash analysis (LFA). To measure the mechanical strength of PPA/BN composites according to the surface treatment of BN, the storage modulus was tested using a Dynamic mechanical analysis (DMA). All the PPA/BN composites used in the experiment were used for the melt-mixing process, using the optimal temperature and processing method.

## 2. Materials and Methods

### 2.1. Materials

Hexagonal BN (ESK Ceramics/3M, Kempten, Germany) powder with a particle size of 12–15 μm and 0.8–1 nm was used in this study. The PPA was purchased from SK chemicals (Gyeonggi-do, Korea). Sodium hydroxide (NaOH) and ethanol (C_2_H_5_OH) were obtained from Daejung Chemicals (Seoul, Korea). Acetone (CH_3_COCH_3_) and iso-propanol (C_3_H_8_O) were purchased from Duksan Science (Gyeonggi-do, Korea). The DI water used in the experiment was directly purified and filtered through the equipment.

### 2.2. Surface Modification of BN

BN particles were dissolved in 5 M NaOH at 120 °C for 24 h to attach hydroxide functional groups to the BN surface. Since BN particles have relatively few surface functionalities, most of the BN particles that reacted in the hot NaOH solvent were submerged in the solvent, but some floated on the NaOH solvent to form a foam. After hydroxide modification, which separated BN and BN-OH, the particles were rinsed with deionized (DI) water and filtered several times to achieve a neutral pH. The resulting NaOH-treated BN particles were dried in a furnace at 80 °C for 24 h and stored in a desiccator [16].

### 2.3. Exfoliation of BNs

**Scheme 1:**
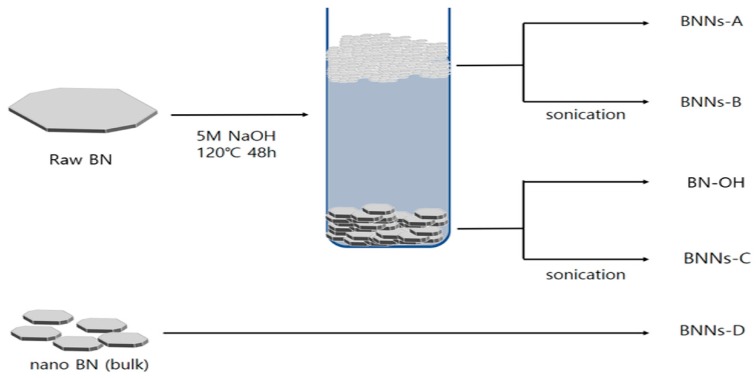
A schematic diagram of boron nitride (BN) surface treatment and Boron nitride nanosheets (BNNs–X).

The exfoliation of BN was produced by three different methods. Firstly, in the case of the BN foam, two types of samples were produced according to the presence of additional exfoliation by ultrasonication or not. These fillers are called BNNs–A and BNNs–B, and are illustrated in Scheme 1 to facilitate understanding. Precipitated BN–OH was produced by BNNs–C through separation using ultrasonication. The ultrasonication used in this experiment reacted during 10 h.

### 2.4. Preparation of PPA-Based Composites

PPA/BN composites were prepared by a melt-mixing method using a twine extruder (model BA–11, L/D ratio = 40, Bau Technology, Seoul, Republic of Korea) at specific temperature ranges. PPA, raw-BN, BN–OH, and BNNs–X (BNNs–A, BNNs–B, BNNs–C, and BNNs–D) were dried at 60 °C for 24 h under vacuum prior to melt mixing. The temperatures of the denoted “feeding zone”, “melting zone”, “mixing zone_1”, “mixing zone_2” and “exit die” were 170, 200, 220, 220, and 200 °C, respectively. The feed rate of the materials and the extrusion speed were held constant at 100 g/min and 80 rpm, respectively. The composites were fabricated with the same rate at 100 g/min and 80 rpm, of material supply and extrusion. The composites and pellets were dried to minimize the pores generated during the manufacturing process and dried for 60 °C for 24 h in the vacuum oven. The specimens for thermal diffusivity testing were prepared by pouring the mixture into a mold and placing it into a compression molding machine (model BA–915, Bau Technology, Seoul, Korea) at a pressure of 12 MPa. The mixture was mixed at 180 °C for 2 min and cooled at room temperature for 1 min after injection. After Molding, each analytical device was analyzed after drying at 80 °C for 24 h before measuring.

### 2.5. Charaterization

FT-IR (Thermofisher Scientific, Nivolet iS5, Waltham, MA, USA) and XPS were used to confirm the successful surface modification of BN including aniline trimer. FT-IR spectra were recorded at a resolution of 4.0 cm^−1^ and scanned over a range of 4000–400 cm^−1^. The XPS analysis was performed using a Kα+ Thermofisher Scientific (Seoul, Korea) X-ray spectrophotometer. The cross-sectional images of PPA/BN composites and h-BN, BNNS particles were confirmed by a field emission scanning electron microscope (FE-SEM, Sigma, Carl Zeiss, Oberkochen, Germany). High-resolution transmission electron microscopy (HR-TEM, JEM-3010, Tokyo, Japan) was used to analyze the images of the h-BN and BNNS particles. The method of calculating the thermal diffusion rate was measured through the time scale from the rear to the upper layer where the heat transfer begins, and the thermal conductivity can be derived by using the following equation:

K = α × ρ × C_p_

where K, α, ρ, and C_p_ are the thermal conductivity (W/(m·K)), thermal diffusivity (m^2^/s), density (kg/m^3^), and specific heat capacity (J/(kg·K)) of the composites, respectively. The thermal diffusivity and thermal conductivity of all samples were analyzed by laser flash analysis (LFA) measurement equipment, which was carried out using a Netzsch 467 nanoflash (Garanti Bank, Atatürk Osb, Turkey). The storage modulus of the PPA/BN composites was measured by a dynamic mechanical analysis (DMA, Triton instrument, Trition DMTA) at a constant frequency of 1 Hz.

## 3. Results

**Figure 1:**
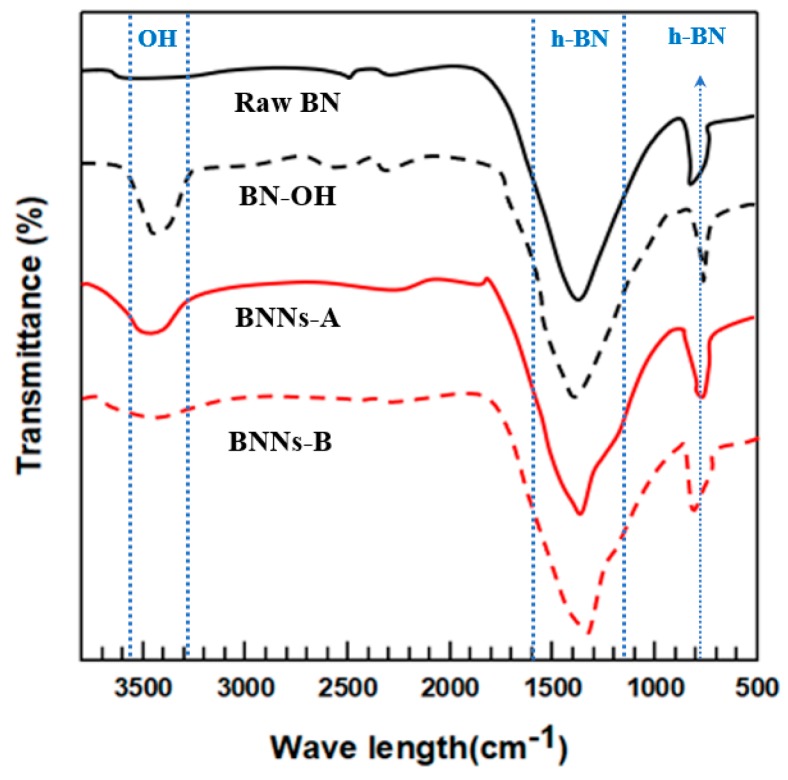
FT-IR spectra of raw BN, BN-OH, BNNs-A and BNNs-B.

**Figure 2:**
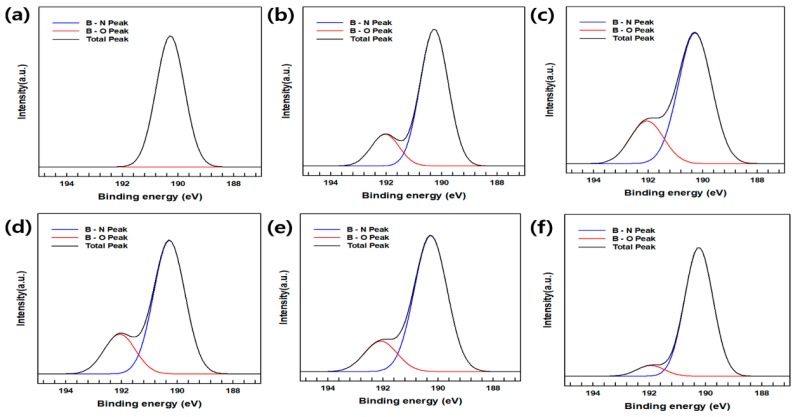
XPS B1s peak of raw BN (**a**), BN–OH (**b**), BNNs–A (**c**), BNNs–B (**d**), BNNs–C (**e**), and BNNs–D (**f**).

As portrayed in Scheme 1, four kinds of BN nanosheet were fabricated according to the presence of BN layer separation and ultrasonic treatment in NaOH solvent. For the exfoliation of the BNNs, 5M NaOH was used to introduce hydroxyl groups onto the surface of the BN. In our recent study, this method was an active method for the introduction of BN hydroxyl groups. Interestingly, after 48 h of surface treatment, the hexagonal BN was divided into two layers of 5 M NaOH solvent [16]. Theoretically, because the density of the hexagonal BN is 2.1 g/cm^3^, it has a density twice as high as that of the Di water, so it must sink in NaOH solution. Nevertheless, some BN formed as foam on the upper part of the NaOH solvent. The BN foam has greater densities than the NaOH solvents, but the BN foam has a wide plate area and lower thicknesses. The exfoliation of BN by sonication is a very popular method because it is simple and safe [17,18]. However, due to the force of sonication, the large plate phase is broken with the separation of BN. In order for the BNN to have excellent properties, it requires a large surface area and a thin thickness. In order to confirm the surface treatment of the BNNs fabricated with the BN foam and sonication, we observed the BN used by FT-IR. The FT-IR spectra of raw BN, BN–OH, BNNs–A, and BNNs–B are shown in Figure 1. Since raw BN without hydroxylated surface treatment are neat states, it confirmed that there was only a simple BN peak at 1400 cm^−1^ and 800 cm^−1^. Additionally, BNNs–A, BNNs–B and the BN–OH surface treated with NaOH all showed a simple peak at 3200–3700 cm^−1^, although there was a slight difference in each –OH peak, and the peak of the BN foam-type BNNs–A was the largest. Since the ceramic flake size was smaller, the wider the area that could be surface-treated, the more successful the surface modification [19]. Furthermore, exfoliation through sonication led to the destruction of the BN particles that had been introduced with the hydroxyl group. Because the edge of the BN formed by the broken was not surface-treated, the separation of BN using sonication had a relatively small amount of –OH functional groups. To further confirm the surface treatment of BN particles, we characterized the samples with XPS. Figure 2 shows the XPS spectra of raw BN, BN–OH, BNNs–A, B, C and D; the latter exhibits various peaks corresponding to BN. The XPS spectra corresponding to the B 1s region of raw BN, BN–OH, and BNNs–A, BNNs–B, BNNs–C are shown in Figure 2; the binding energies ranged from 195 to 187 eV. The raw BN and BNNs-D had a simple B 1s peak, and BNNs–A, BNNs–B, BNNs–C, and BN–OH exhibited multiple features at 192 eV, which were deconvoluted. The peak located at 192 eV represents the B–O bond, which was proportional to the FT-IR data [20,21].

**Figure 3:**
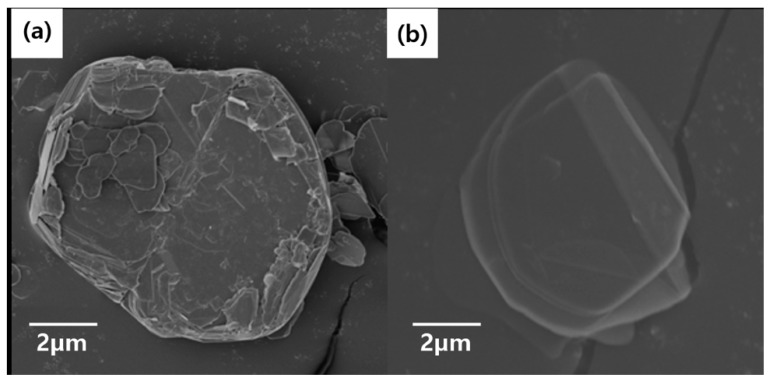
Field emission scanning electron microscope (FE-SEM) image of raw BN particles (**a**), and BN–OH particles (**b**).

**Figure 4:**
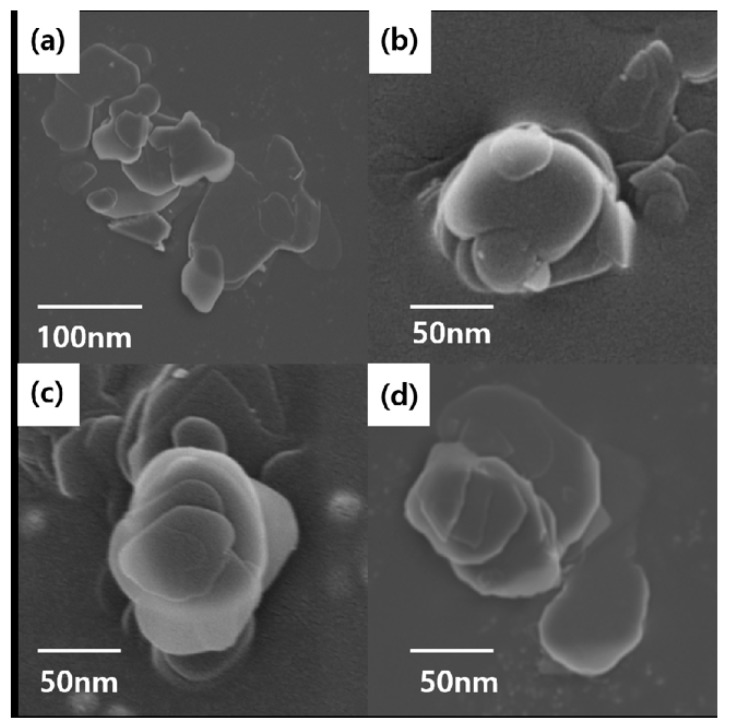
FE-SEM image of used raw BN particles (**a**), BNNs–A particles (**b**), BNNs–B particles (**c**) and BNNs–C particles (**d**).

**Figure 5:**
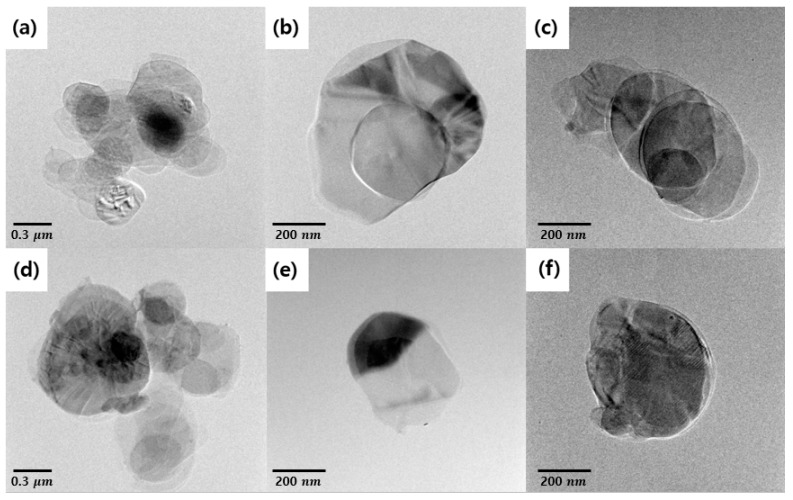
High-resolution transmission electron microscopy (HR-TEM) image of BNNs–A particles (**a**,**b**), BNNs–B particles (**c**,**d**) and BNNs–C particles (**e**,**f**).

**Figure 6:**
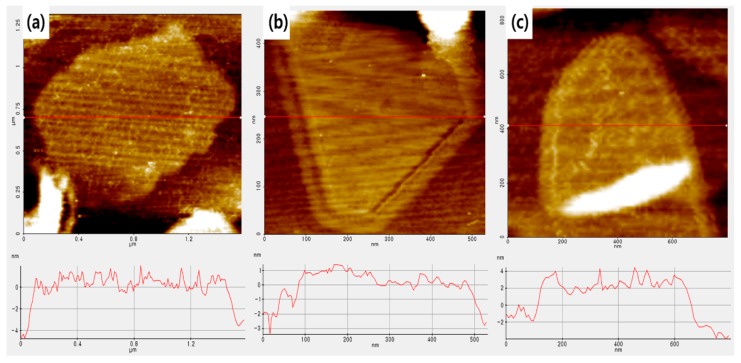
AFM analysis of BNNs–A (**a**), BNNs–B (**b**) and BNNs–C (**c**).

After confirming the successful surface treatment of BN Foam, we analyzed the particle sizes of BNNs–A, BNNs–B, and BNNs–C using FE-SEM, TEM, and AFM as shown in Figure 3. A particle image of untreated raw BN and surface-modified BN–OH was confirmed. After checking the raw BN particles, it was evident that BN plates of various sizes were placed on the BN surface of the micro-sized BN. However, the surface of the BN–OH particles treated by NaOH was relatively sleeker than that of the raw BN. Because BN–OH had been reacting for a long time at a high temperature, it was partly peeled off by the surface-treated hydroxyl group. Thus, BN particles that have been surface-treated and deviate from the micro BN plates float in the NaOH solvent and form a foam. Figure 4 shows FE-SEM images for comparing the shapes of BNNs–A, B, C and D. BNNs–A, BNNs–B, and BNNs–C were all found to have a thin plate-like structure, except for the unbaked BNNs–D in bulk form. BNNs–A had the broadest plate form, but all BNNs–X were aggregated and it was difficult to accurately compare the size and thickness of the plate using only the FE-SEM image. Therefore, for precise comparison and analysis, BNNs were compared using TEM and AFM, and were analyzed through a grid after being dispersed in a solvent. BNNs–D, which was relatively thick, was not suitable for the analysis of TEM and AFM, and was therefore excluded from the measurement. Figure 5 is a TEM image of BNNs–A, BNNs–B, and BNNs–C. The BNNs were completely transparent to an electron beam image due to the extremely thin shape. All the BNNs were identified as having multiple layers rather than a single layer. This means that the more transparent the BNNs, the thinner the layers in the pile. First, for BNNs–A, a thin BN layer structure with a large area was identified. By comparison, BNNs–B and BNNS–C had a relatively smaller surface area than BNNs–A. Because of the energy of the ultrasonic treatment used for the BN exfoliation, the BN particles were partially destroyed. BNNs–B were thinner and smaller compared with BNNs–C using micro-sized BN particles because of the additional separation of foam-type BNNs–A with ultrasonication. However, due to the energy of ultrasonication, the surface area was greatly reduced compared with BNNs–A. BNNS–A, BNNs–B, and BNNs–C were measured using AFM for a more accurate measurement of the width and thickness of each nanosheet. Each nanosheet was measured with AFM through noncontact mode, and was compared with a well dispersed single plate for more accurate measurement. In Figure 6, the thickness and width of each of the three types of BNNs can be seen. BNNs–B and BNNs–C prepared by ultrasonication were 3.5 nm and 6.2 nm thick, respectively, and very thin nanosheets were fabricated. Surprisingly, the thickness of the foam-type BNNs–A, which was prepared only with NaOH treatment without using ultrasonication, was 4.5 nm thick, similarly to other BNNs prepared by ultrasonication (shown below), including the AFM data in Figure 6. As a result, the BN foam without ultrasonication could produce nanosheets with a high aspect ratio, as well as introduce hydroxyl groups through the NaOH solvent.

**Figure 7:**
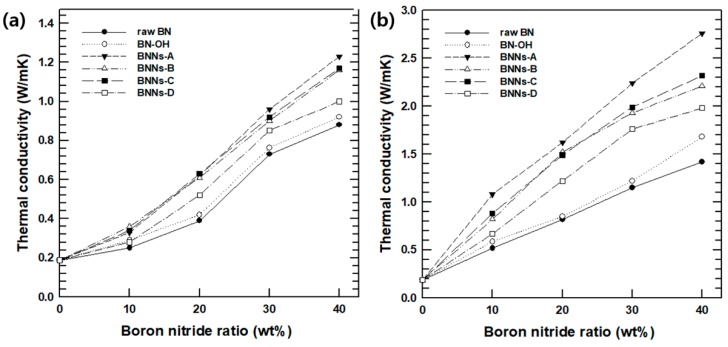
Thermal conductivity from LFA data of raw PPA, raw BN/PPA, BN–OH/PPA BNNs–X/PPA, through plane(**a**), and in-plane (**b**).

After confirming the successful exfoliation of BNNs, we measured the change in mechanical strength and thermal conductivity of PPA through a combination of different methods of BNNs–X with PPA. The thermal conductivity of the PPA/BN composites according to the weight fraction (10–40%) of each raw BN, BN–OH, and BNNs–X are shown in Figure 7a. As for through-plane thermal conductivity, the thermal conductivity of the raw PPA was approximately 0.18 W/(m·K), and the through-plane thermal conductivity of the BNNs-A composites increased from 2.89 W/(m·K), which was 44% that of PPA/raw–BN. Other BNNs composites also showed an increased thermal conductivity. Moreover, the successful peeling exfoliations of BNNs–A, BNNs–B, and BNNs–C composites showed an excellent increase in thermal conductivity. These results were the same for in-plane thermal conductivity. As for in-plane thermal conductivity, as shown in Figure 7b, PPA/BNNs–A, which has the highest in-plane thermal conductivity, showed an approximately 2.31-fold higher thermal conductivity than that of PPA/BN combined with conventional raw BN. As such, exfoliation BNNs with PPA composites could lead to a higher thermal conductivity than the micro-size BN [22,23]. Since the successfully peeled BN nanosheets had excellent dispersibility among the PPA matrix, the heat flowed inside the composite much more smoothly. Moreover, in the case of BNNs–A, which had the highest hydroxyl group introduction, the interfacial affinity with the PPA matrix increased, which reduced the air gap between the BN particles and the PPA matrix, and formed much better composites.

**Figure 8:**
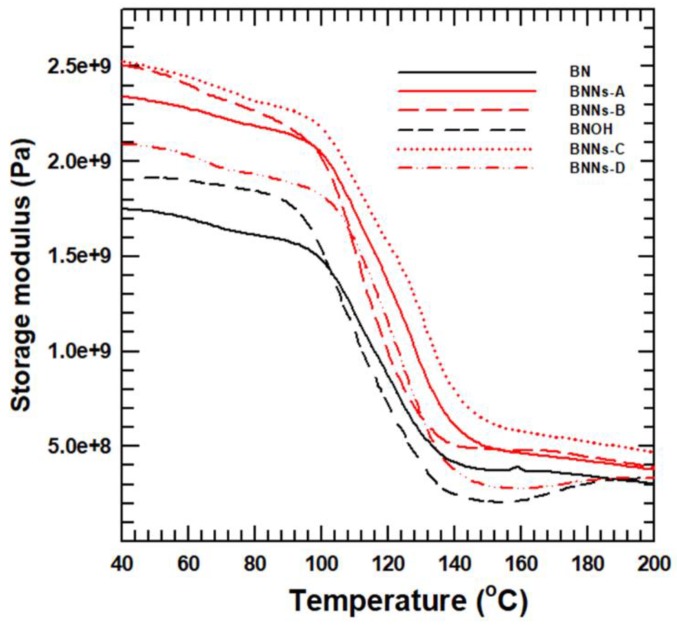
DMA analysis of the raw PPA and the BN/PPA, BN–OH/PPA and BNNs–X/PPA composites.

The mechanical strength of the PPA composite was analyzed by DMA, which was measured by the difference of storage modulus. The temperature range of 30–200 °C was measured at 1 Hz, which is sufficiently used for the range of PPA matrix shown in Figure 8. At any given temperature, the storage modulus of the PPA composites increased in the following order: raw PPA < PPA/raw–BN < PPA/BN–OH < PPA/BNNs–D < PPA/BNNS–A < PPA/BNNs–C < PPA/BNNS–B. This result was slightly different from the LFA data, with the highest exfoliation BNNs–B having the best storage modulus. In particular, BN nanosheets have the largest surface area in contact with PPA matrix, which shows the greatest improvement in storage modulus. One of the reasons is that PPA matrix on the surface of BN particles loses its liquidity, which causes an improvement in the storage modulus of the composite. Therefore, BN nanosheet composites with the widest surface area of mass ratio have the best storage modulus. Finally, the composite with the thinnest, finest strips, BNNs-B–had the best storage modulus.

**Figure 9:**
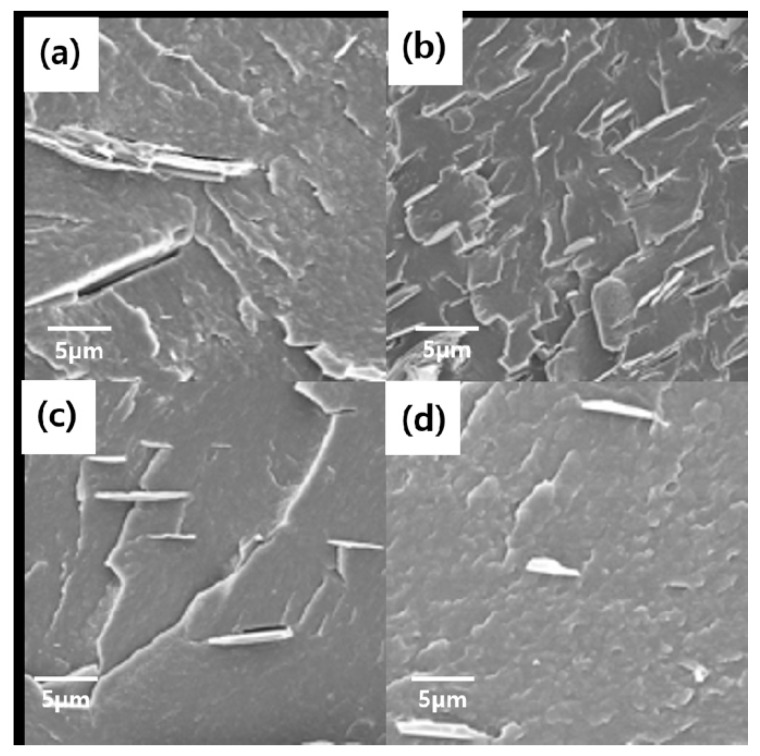
Cross-section image of BN/PPA composites raw BN (**a**), BNNs–A (**b**), BNNs–B (**c**) and BNNs–C (**d**).

In Figure 9, the surface of BN particles and the PPA matrix have a low interfacial affinity, which indicates that an air gap exists between the surface of pure BN particles and the PPA matrix. It is well known [16] that the surface of BN is highly friendly with the other BN particles, and it is known that physical and chemical treatment is very difficult. However, in the case of the PPA/BN–OH and PPA/BNNs–X composites, it is evident that the interfacial affinity between PPA and BNNs was excellent due to the surface treatment of hydroxyl groups. Furthermore, BNNs composites with PPA can be well dispersed in the PPA matrix without destroying the particles.

## 4. Discussion

BN is a two-dimensional material with a high thermal conductivity. However, because it has a layered structure, the anisotropic property can be maximized when peeled off and manufactured in the form of nanosheets. In this study, we made BNNS through a simple solution reaction, which was applied to polymer composites and showed an effective thermal conductivity improvement. The method established in this study can be used for future research using BNNS.

## 5. Conclusions

In this study, we successfully succeeded in stripping BN in nanosheets using only a simple introduction of a hydroxyl group without using ultrasonication. This method minimizes the destruction of BNNs and confirms successful hydroxyl groups surface treatment. The effects of the surface treatment of BN with NaOH were investigated. The chemical functionalities of raw–BN, BN–OH, and BNNs–X were revealed by FTIR and XPS. To confirm successful BNNs–X exfoliations, FE–SEM, HR–TEM, and AFM were used to analyze the width and thickness of each BNNs–X. The in-plane thermal conductivity and through-plane thermal conductivity of the PPA/BNNs–A composites were the highest. The highest value in-plane of thermal conductivity was 2.89 W/(m·K), which is a 16-fold increase over pristine PPA. The mechanical properties of the resulting composites were tested to determine the storage modulus used by DMA. The storage moduli of the composites with PPA/BNNs–B were higher than those of PPA/BNNs–A because it was peeled off with the smallest size, and the fluidity of the PPA matrix was minimized. Furthermore, BNNs–X exfoliation through the hydroxide introduction showed an improved interfacial affinity with PPA compared to the conventional BN, thereby minimizing the air gap and improving the dispersibility.
